# The Complete Mitochondrial Genome of *Mindarus keteleerifoliae* (Insecta: Hemiptera: Aphididae) and Comparison with Other Aphididae Insects

**DOI:** 10.3390/ijms161226219

**Published:** 2015-12-17

**Authors:** Yuan Wang, Jing Chen, Li-Yun Jiang, Ge-Xia Qiao

**Affiliations:** 1 Key Laboratory of Zoological Systematics and Evolution, Institute of Zoology, Chinese Academy of Sciences, Beijing 100101, China; wangyuan0330@163.com (Y.W.); chenjing@ioz.ac.cn (J.C.); jiangliyun@ioz.ac.cn (L.-Y.J.); 2 College of Life Sciences, University of Chinese Academy of Sciences, Beijing 100049, China

**Keywords:** aphid, *Mindarus keteleerifoliae* Zhang, mitogenomes, tRNA isomerism, phylogenetic relationships

## Abstract

The mitogenome of *Mindarus keteleerifoliae* Zhang (Hemiptera: Aphididae) is a 15,199 bp circular molecule. The gene order and orientation of *M. keteleerifoliae* is similarly arranged to that of the ancestral insect of other aphid mitogenomes, and, a tRNA isomerism event maybe identified in the mitogenome of *M. keteleerifoliae*. The *tRNA-Trp* gene is coded in the J-strand and the same sequence in the *N*-strand codes for the *tRNA-Ser* gene. A similar phenomenon was also found in the mitogenome of *Eriosoma lanigerum*. However, whether tRNA isomers in aphids exist requires further study. Phylogenetic analyses, using all available protein-coding genes, support Mindarinae as the basal position of Aphididae. Two tribes of Aphidinae were recovered with high statistical significance. Characteristics of the *M. keteleerifoliae* mitogenome revealed distinct mitogenome structures and provided abundant phylogenetic signals, thus advancing our understanding of insect mitogenomic architecture and evolution. But, because only eight complete aphid mitogenomes, including *M. keteleerifoliae*, were published, future studies with larger taxon sampling sizes are necessary.

## 1. Introduction

Generally, the insect mitochondrial genome (mitogenome) consists of a circular, two-stranded genome, 14,000–19,000 bp in length, which contains 37genes, including 13 protein coding genes (PCGs), cytochrome c oxidase subunits 1–3 (*cox1*, *cox2* and *cox3*), cytochrome b (*cob*), subunits 6 and 8 of the ATPase (*atp6* and *atp8*), NADH dehydrogenase subunits 1–6 and 4L (*nad1*–*6* and *nad4L*), two ribosomal RNA genes encoding the large and small subunit rRNAs (*rrnL* and *rrnS*) and 22 transfer RNA (tRNA) genes. An A+T-rich region, also named the control region, containing initiation sites for transcription and replication was also found in the insect mitogenome [1,2,3,4]. Mitogenomes have been widely used in studies regarding insect molecular systematics, phylogeography and population genetics [5,6,7].

Aphidinea belong to the order Hemiptera, which contains three families: Aphididae, Adelgidae and Phylloxeridae [8]. This insect group includes more than 5000 species worldwide, and the family Aphididae contains most of the species in 25 subfamilies [9]. Aphids have many intrinsically interesting characteristics, such as complex life cycles, pleomorphism and polymorphism, harboring different endosymbionts, inducing diverse galls on host plants and differentiation of social behavior [10].Therefore, all these characteristics make aphids anappropriate model for ecological and evolutionary studies [11].

Out of numerous aphids worldwide, only few have a complete or near-complete mitogenome currently available in GenBank (Table 1). Especially, except for the Aphidinae, the data for other subfamilies are quite rare. In 2014, we reported the mitogenome of *Cervaphis quercus* Takahashi (Aphididae: Greenideinae), which was the first and only mitogenome of aphids not belonging to the Aphidinae so far [12].

**Table 1:** The mitochondrial genomes of aphids.

Category		Species	Length (bp)	GenBank No.	Reference
Aphidida**e**	Aphidinae	*Schizaphis graminum*	15,721	NC_006158	Thao *et al.*, 2004 [13]
		*Acyrthosiphon pisum*	16,971	NC_011594	IAGC, 2010 [14]
		*Diuraphis noxia*	15,784	NC_022727	Zhang *et al.*, 2014 [15]
		*Sitobion avenae*	15,180	NC_024683	Zhang *et al.*, 2014 [16]
		*Cavariella salicicola*	16,317	NC_022682	Wang *et al.*, 2013 [17]
		*Aphis gossypii*	15,869	NC_024581	Zhang *et al.*, 2014 [18]
	Greenideinae	*Cervaphis quercus*	15,272	NC_024926	Wang *et al.*, 2014 [12]
	Mindarinae	*Mindarus keteleerifoliae*	15,199	KP722576	This study
Phylloxeridae		*Viteus vitifoliae* *	12,349	DQ021446	Direct submission

* Nearly complete.

The subfamily Mindarinae was considered a relatively age-old subfamily in Aphididae [19]. Species of this subfamily are conifer-feeding aphids, where the apterae with head fused with pronotum have three-facetted eyes, well-developed dorsal wax glands, and a tongue-shaped cauda [20].This subfamily only contains one genus, *Mindarus* Koch, which has extant species and eight fossil species worldwide [9]. *Mindarus keteleerifoliae* Zhang is a Chinese endemic species from the Hengduan Mountains [21]; and it mainly infests the leaves and young shoots of *Keteleeria* Carr. plants, an endemic plant group in East Asia.

Therefore, in the present study, we sequenced and annotated the complete mitogenome of *M. keteleerifoliae*, which represent the subfamily Mindarinae. Aphids of *M. keteleerifoliae* are harmful to old coniferous trees because they cause leaves to change color and die. Our results identified a tRNA isomer in the aphid mitogenome. Furthermore, the *M. keteleerifoliae* mitogenome was compared with mitogenomes from other aphids, thus increasing our understanding of aphid phylogeny and evolution.

## 2. Results and Discussion

### 2.1. Genome Organization and Composition

**Figure 1:**
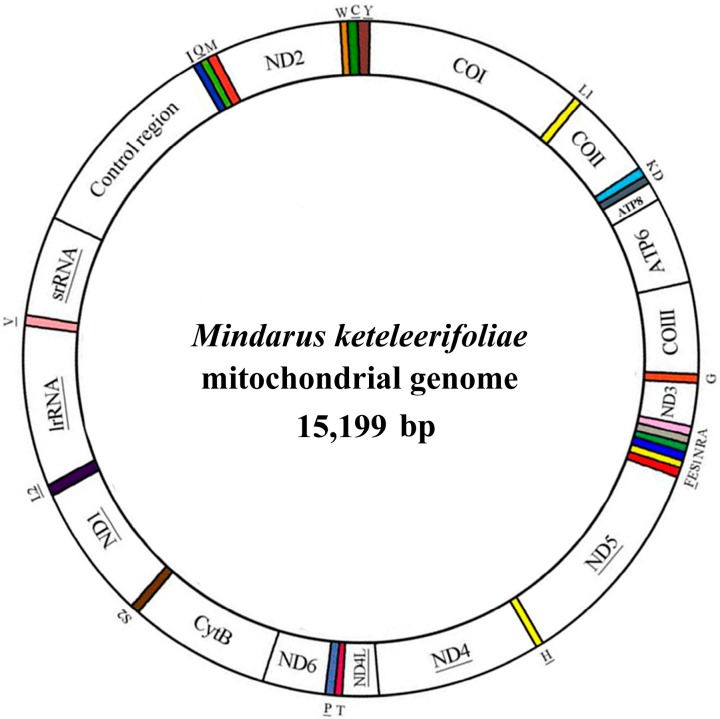
Circular map of the *Mindarus keteleerifoliae* mitogenome. Gene names not underlined indicate the direction of transcription in the major strand, and underlined names indicate the direction of transcription in the minor strand. The transfer RNAs (tRNAs) are denoted by the colored blocks and are labeled according to the single-letter amino acid codes.

The complete mitogenome of *M. keteleerifoliae* is a double-stranded plasmid with 15,199 bp containing 13 PCGs, 22 tRNA genes, 2 rRNA genes, and a control region (Figure 1). Twenty-three genes were transcribed on the majority strand (J-strand), whereas the other fourteen were coded on the minority strand (N-strand). This mitogenome sequence was then submitted to GenBank (No. KP722576). These genes were arranged in the same order as the inferred insect ancestral mitogenome [3], *Drosophila yakuba* [22]. Currently, *M. keteleerifoliae* has the shortest mitogenome length in aphids (Table 1). The length variation was conserved in PCGs, tRNAs, *rrnS* and *rrnL*, which was due to variation in the mainly intergenic spacers, such as the control region and repeat region [12,15]. In *M. keteleerifoliae*, 10 overlaps (a total of 33 bp) between adjacent genes were detected (Figure 1, Table 2). The *atp8-atp6* overlap often exists in insect mitogenomes and is 7 bp [23]. However, exceptions to this overlap were found in aphids (a 20-bp overlap in most aphids, and a 14-bp overlap between *atp6* and *atp8*in *Diuraphis noxia*) [12,15]. Actually, there are many exceptions in other reported insects, such as a 19-bp overlap in the honeybee (*Apis mellifera*) [24] and a 244-bp long spacer in a hymenopteran (*Evania appendigaster*) [25]. Therefore, Lavrov’s 7 bp hypothesis will be challenged with the increasing data offered by this mitogenome analysis.

**Table 2:** Organization of the *Mindarus keteleerifoliae* mitochondrial genome.

Gene	Strand	Position	Anticodon	Size (bp)	Start Codon	Stop Codon	Intergenic Nucleotides *
*cox1*	J	1–1531	–	1531	ATA	T	−1
*tRNA-Leu*	J	1531–1596	TAA	66	–	–	3
*cox2*	J	1600–2271	–	672	ATA	TAA	–
*tRNA-Lys*	J	2272–2344	CTT	73	–	–	7
*tRNA-Asp*	J	2352–2419	GTC	68	–	–	17
*atp8*	J	2437–2586	–	150	ATA	TAA	−20
*atp6*	J	2567–3220	–	654	ATT	TAA	
*cox3*	J	3221–4006	–	786	ATG	TAA	−1
*tRNA-Gly*	J	4007–4068	TCC	62	–	–	−3
*nad3*	J	4066–4422	–	357	ATA	TAA	–
*tRNA-Ala*	J	4423–4487	TGC	65	–	–	−1
*tRNA-Arg*	J	4487–4555	TCG	69	–	–	−1
*tRNA-Asn*	J	4555–4623	GTT	69	–	–	−1
*tRNA-Ser*	J	4623–4684	TCT	62	–	–	6
*tRNA-Glu*	J	4691–4764	TTC	74	–	–	3
*tRNA-Phe*	N	4768–4837	GAA	70	–	–	–
*nad5*	N	4838–6508	–	1671	ATT	TAA	–
*tRNA-His*	N	6509–6574	GTG	66	–	–	–
*nad4*	N	6575–7883	–	1309	ATA	T	5
*nad4L*	N	7889–8179	–	291	ATA	TAA	–
*tRNA-Thr*	J	8180–8245	TGT	66	–	–	–
*tRNA-Pro*	N	8246–8316	TGG	71	–	–	1
*nad6*	J	8318–8812	–	495	ATT	TAA	−1
*cob*	J	8812–9927	–	1116	ATG	TAA	−2
*tRNA-Ser*	J	9926–9990	TGA	65			10
*nad1*	N	10001–10936	–	936	ATT	TAA	–
*tRNA-Leu*	N	10937–11001	TAG	65	–	–	–
*rrnL*	N	11002–12267	–	1266	–	–	1
*tRNA-Val*	N	12269–12330	TAC	62	–	–	3
*rrnS*	N	12334–13098	–	765	–	–	–
control region	–	13099–13785	–	687	–	–	–
*tRNA-Ile*	J	13786–13851	GAT	66	–	–	3
*tRNA-Gln*	N	13855–13920	TTG	66	–	–	29
*tRNA-Met*	J	13950–14017	CAT	68	–	–	–
*nad2*	J	14018–14995	–	978	ATA	TAA	–
*tRNA-Trp*	J	14996–15063	TCA	68	–	–	−2
*tRNA-Cys*	N	15062–15127	GCA	66	–	–	4
*tRNA-Tyr*	N	15132–15198	GTA	67	–	–	1

* indicates the intergenic spacer, negatives indicate the nucleotide number of gene overlap.

The nucleotide composition of *M. keteleerifoliae* is typically A+T biased with 84.52%and is slightly A skewed (0.06) and strongly C skewed (−0.23) (Supplementary Material, Table S1). Similar patterns of nucleotide composition were also found in other aphid species and gathered into a cluster [26], representing high A+T content in Hemipteran species. Analysis of the base composition at each codon position of the concatenated 13 PCGs suggested that the A+T content of all codon positions is above 80% (Supplementary Material, Table S1). In the first codon position, the strongest bias toward T was (48.0%), while the third codon position had the highest G+C content (19.0%). Actually, aphids with A+T rich and G-deficit mitogenomes were similar to other insects [3]. GC content strongly determines codon bias as well as mutational selection and pressure at the genome level in the prevailing theories of neutral mutations[27].The low GC content found in aphid population genetic studies may indicate a potential explanation for the slow mutation rates observed commonly in aphid mtDNA sequences [28,29].

### 2.2. Protein-Coding Genes

The mitogenome of *M. keteleerifoliae* includes the 13 PCGs that are present in animal mitogenomes and their arrangement orders are similar to the standard order of insect [3]. In the *M. keteleerifoliae* mitogenome, the start codon is one of the typical ATN codons, four (*atp6*, *nad1*, *nad5* and *nad6*) with ATT, two (*cob*and *cox3*) with ATG, and the remainder with ATA (Table 2). The most common TAA termination codons were detected in 11 PCGs (*cox2*, *cox3*, *cob*, *atp6*, *atp8*, *nad1*, *nad2*, *nad3*, *nad4L*, *nad5* and *nad6*). *Cox1* and *nad4*, the remaining two, had incomplete termination codons with T (Table 2). In the mitogenomes of most insects, partial stop codons are common [1], including the currently sequenced aphid species [12,15,17].

There are 3637 amino-acid-coding codons that were calculated for codon usage of the *M. keteleerifoliae* mitogenome. Approximately the same codon numbers were found in *Acyrthosiphon pisum* (Harris) (3637) and *Schizaphis graminum* (Rondani) (3638). The five most abundant codons are UUU (Phe), UUA (Leu), AUU (Ile), AUA (Met) and AAU (Asn) (Supplementary Material, Table S2), and reflect the A+T bias. Cystine is the least frequent as in other aphids [12,15]. The third codon positions have the strongest A+T bias (Supplementary Material, Table S2).

### 2.3. tRNA and rRNA

**Figure 2:**
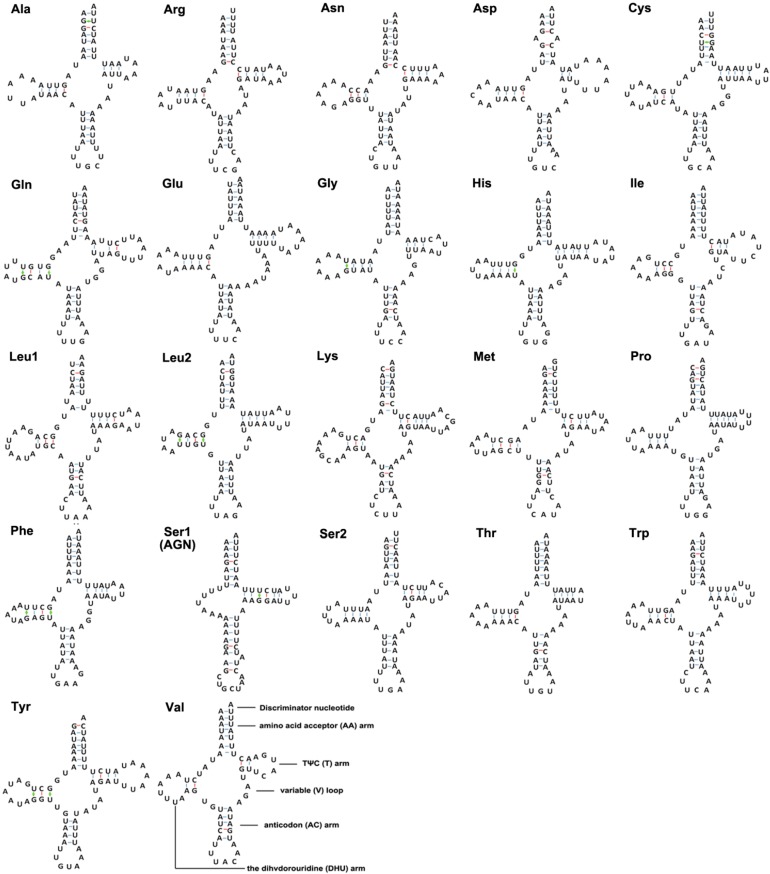
The inferred secondary structure of the 22 transfer RNAs (tRNAs) in the *Mindarus keteleerifoliae* mitogenome. The tRNAs are labeled with the abbreviations of their corresponding amino acids. Dashed line (−) indicates Watson–Crick base pairing and (+) indicates G–U base pairing.

In the *M. keteleerifoliae* mitogenome (Table 2), all 22 typical animal tRNA genes with length from 62 to 74 bp were found, and 20 were determined using the tRNAscan-SE [16]. By comparison with currently published aphid mitogenomes, the *tRNA-Asn* and *tRNA-Ser*(AGN) genes were determined [12,13,15,17]. The typical clover-leaf structure was predicted in only 21 of the 22 mitochondrial tRNAs since the *tRNA-Ser*(AGN) gene included a DHU replacement loop instead of the typical DHU arm, and could not form a stem-loop structure in the TΨC arm (Figure 2). In many arthropod mitogenomes, this is a common feature [1].

Based on the secondary structure, in the *M. keteleerifoliae* tRNAs, a total of 13 G–U weak base pairs were found, which form weak bonds, located in the AA stem (2 bp), the T stem (1 bp) and the DHU stem (10 bp) (Figure 2). Most of the mismatched nucleotides were G–U pairs, which form weak bonds in tRNAs and non-canonical pairs in tRNA secondary structures similarly to other aphids [12,17].

The boundaries of rRNA genes were implemented from the alignment with other aphid species [12,13,17]. The *rrnL* of *M. keteleerifoliae* was located between *tRNA-Leu*(CUN) and *tRNA-Val*, and *rrnS* resided between *tRNA-Val* and the control region similarly to other insects (Figure 1). The large ribosomal gene (*rrnL*) of *M. keteleerifoliae*was1266 bp, and has an AT content of 85.6%. The small ribosomal gene (*rrnS*) was 765 bp, and has an AT content of 84.4% (Supplementary Material, Table S1). The identified AT contents were same to those reported species in other hemipterans [30,31].

### 2.4. tRNA Isomerism

**Figure 3:**
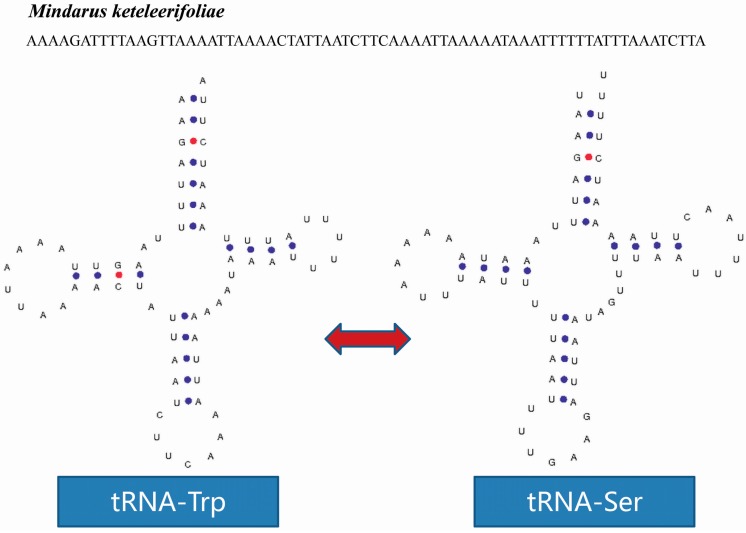
The tRNA isomer of *Mindarus keteleerifoliae*. The blue and red dots indicate Watson–Crick base pairing.

**Figure 4:**
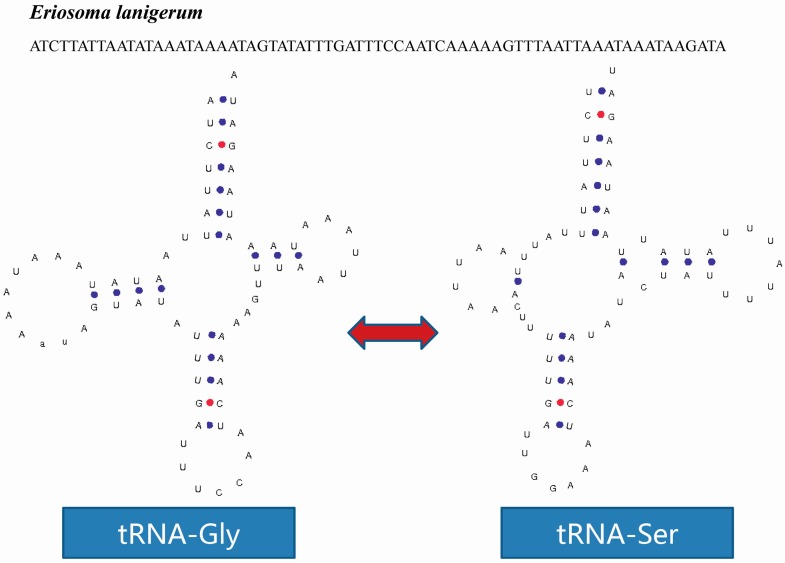
The tRNA isomer of *Eriosoma lanigerum*. The blue and red dots indicate Watson–Crick base pairing.

A tRNA isomer may be discovered in the mitogenome of *M. keteleerifoliae*. The *tRNA-Trp* gene is coded in the J-strand; however, the same sequence in the N-strand codes for the *tRNA-Ser* gene (Figure 3). This phenomenon was also found in the mitogenome of *Eriosoma lanigerum* (our unpublished data): the *tRNA-Gly* gene and *tRNA-Ser* occurred as atRNA isomer (Figure 4).

Therefore, both tRNAs were *tRNA-X* and *tRNA-Ser* isomers. First, the spatial structure of the two tRNA isoforms was stable in terms of chemical free energy. However, in the *tRNA-Ser* gene of *M. keteleerifoliae*, the acceptor arm lost a pair of hydrogen bonds; and in the *tRNA-Ser* gene of *Eriosoma lanigerum* (Hausmann), the D loop and TΨC loop arms lost two pairs of hydrogen bonds. The number of hydrogen bonds retained a low free energy and maintained stable spatial structure, leading to the formation of the typical cloverleaf secondary structure. Second, from the four types of amino acid codons involved in the two isomer phenomenon, the codons UCA and UGA had relatively higher usage frequencies in *M. keteleerifoliae* (Supplementary Material, Table S2); the usage frequency the codon UCC in *Eriosoma lanigerum* was relatively weak but not zero, and the frequency for GGA was relatively higher. After analyzing these two points, the tRNA isomers in aphids may exist (rather than resulting from the coincidence of sequencing results). These predicted tRNA structures cannot discern their function, but several studies have confirmed that tRNA isomers exist and are functional [32]. In aphids, determining whether the tRNA isomers exist also need further study.

### 2.5. Non-Coding Regions

Sixteen non-coding regions, with a total of 770 bp, were interspersed throughout the *M. keteleerifoliae* mitogenome (Table 2). As in typical insect mitogenomes, the *M. keteleerifoliae* mitogenome includes one large non-coding region, identified as the control region.

**Figure 5:**
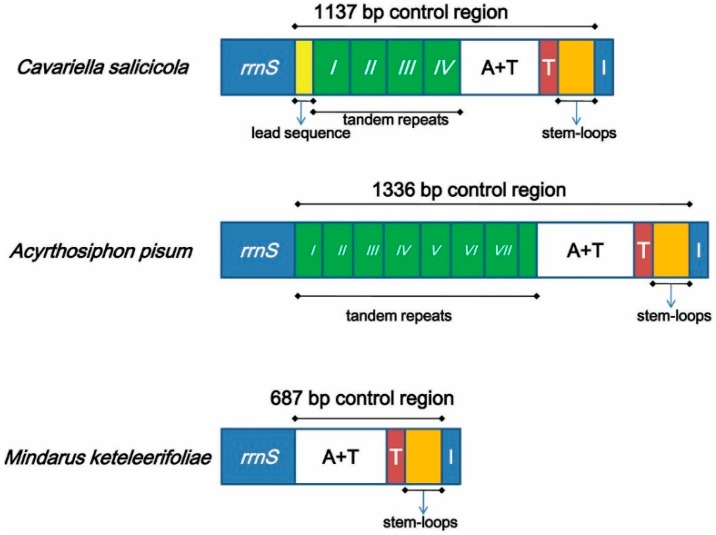
Control region organization in aphid mitogenomes. The lead region is in a yellow box; the green boxes with Roman numerals indicate the tandem repeat units; A+T represents a high A+T content region; red boxes refer to the poly-thymidine stretch; orange boxes indicate the stem-loop region.

In *M. keteleerifoliae*, the control region was rich in A+T (88.9%) and located downstream of *rrnS*. This region commonly contains replication origins in both invertebrates and vertebrates [2,3]. Meanwhile, the control region had a higher A+T content than the whole majority strand of all reported aphid mitogenomes [12,15,17]. The lengths of the control regions in aphid mitogenomes are variable, as shown by the control region of *M. keteleerifoliae* with a length of687 bp, which is approximately half the length of *A. pisum* (1336 bp)and *Cavariella salicicola* (Matsumura) (1137 bp), although similar to *C. quercus* (657 bp), *S. graminum* (682 bp) and *Diuraphis noxia* (Kurdjumov) (664 bp). These differences may cause their various structural patterns. Only two species of Aphidinae contained tandem repeat sequences: *A. pisum* and *C. salicicola*[17]. The control regions of *A. pisum* and *C. salicicola* can be divided into four parts (Figure 5): a region composed of complete tandem repeats and a partial copy of the anterior repeat unit; an A+T rich zone; a conserved PolyT stretch; and a stem-loop region at the end of the control region [12,17]. However, the control regions of other aphid species also have the conserved structural pattern found in *M. keteleerifoliae* with three parts (Figure 5): the AT-rich region, the stem-loop structure, and the PolyT stretch regions, which were proposed as a widespread feature in Aphididae [12].

The repeat region between *trnE* and *trnF* is an interesting feature of the aphid mitogenomes [17]. This unique region contains serially variable numbers of tandem repeats, but this repeat region was not found in the mitogenome of *M. keteleerifoliae*. This new evidence implies that the repeat region may be typical for Aphidinae but not of other subfamilies in Aphididae [12]. So, we thought that the repeat region within Aphidinae is lineage specific and occurred from independent evolutionary events.

### 2.6. Phylogenetic Analyses

**Figure 6:**
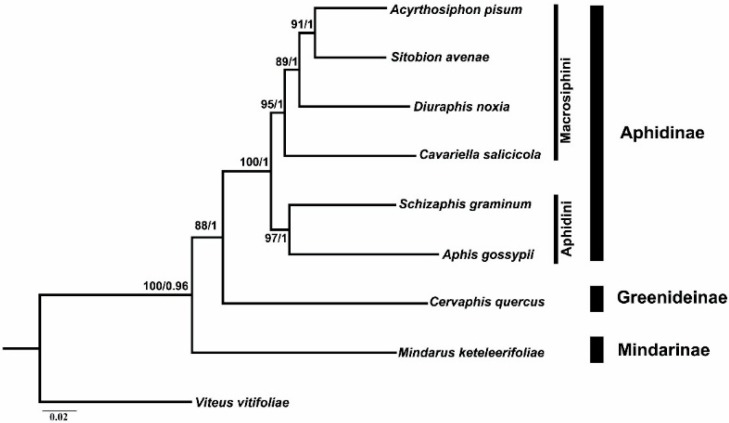
ML and BI Phylogenetic tree inferred from aphid mitogenome sequences. The node support values are the bootstrap (BS) values and the Bayesian posterior probabilities (BPP).

For the phylogenetic analysis, the newly sequenced *M. keteleerifoliae* was combined with the mitogenome sequences of seven aphid species. The phylogenetic trees generated from ML analyses and Bayesian inferences showed similar topologies (Figure 6). The monophyly of Aphidinae, Greenideinae and Mindarinae was recovered in different analyses and well supported. *M. keteleerifoliae* was used as the representative of Mindarinae and located in the basal position of the Aphididae. This result suggests that the Mindarinae aphids are the ancient species of Aphididae and were conifer feeders able to retain an ancestral host relationship with gymnosperms [33]. Meanwhile, some evidence of fossil characteristics also supported the position of Mindarinae [19,34]. Within the Aphidinae subfamily, the monophyly of Aphidini and Macrosiphini had statistically high values, and is similar to the traditional taxonomic views based on morphology [35], and the results based on previous molecular phylogenetic studies [36,37].

## 3. Materials and Methods

### 3.1. Experimental Sample

About 50 individuals of *M. keteleerifoliae* were collected from Kunming, Yunnan Province in 2006. The specimens for slide mounting were preserved in 75% ethanol, and samples for molecular research were stored in 95% ethanol at −20 °C before the DNA extraction. Specimen examination was conducted by Gexia Qiao with a Leica microscope (Leica DM2500, Wetzlar, Germany) based on identified specimens, monographs [21], and the original morphological descriptions. All voucher specimens and samples were deposited in the National Zoological Museum of China at the Institute of Zoology, Chinese Academy of Sciences, Beijing, China (NZMCAS).

### 3.2. DNA Extraction, Amplification and Sequencing

Using the DNeasy Tissue Kit (QIAGEN, Hilden, Germany), total DNA was extracted from single aphids preserved in 95% ethanol following the protocols. Short and long PCR cycles were used to amplify the whole mitogenome of *M. keteleerifoliae.* Short amplicons reactions were amplified using universal primers (some modified), and long amplicons were amplified using specific primers according to the fragments of the short amplicons similar to our previous study [12]. The primers were designed according to the conserved regions by the program Primer premier 5.0. In this study, all the primers used were synthesized by Invitrogen Biotech (Beijing, China) and are listed in Table S3 [38,39,40,41]. Short amplicons (sequence length <1.5 k) were amplified at the following settings with *Taq* DNA polymerase (TransGen Biotech, Beijing, China): 95°C for 3 min; 35 cycles of 92°C for 1 min, 48–55°C (depending on primer pairs) for 1 min and 72°C for 2 min. A final extension step of 10 min was at 72°C after cycling. Long amplicons (sequence length >1.5 k) were amplified under the following cycling conditions with the High Fidelity (HiFi) *Taq* DNA polymerase (TransGen Biotech, Beijing, China): 2 min at 92 °C, 10 cycles (10 s at 92 °C, 30 s at 50–55°C (depending on primer pairs), and 4–8 min at 68 °C), 20 cycles (10 s at 92 °C, 30 s at 50–55 °C, and 4–8 min at 68 °C with an additional 30 s per cycle), and a final prolonged elongation of 10 min at 72 °C. PCR products were detected by electrophoresis on a 1% agarose gel and purified using the *EasyPure* PCR purification Kit (TransGen Biotech, Beijing, China). All short PCR fragments were directly sequenced for both strands. Long PCR fragments were cloned into the pMD19-T sequencing vector (TaKaRa, Dalian, China) and sequenced using the primer walking strategy. Sequencing reactions were implemented by the BigDye Terminator v3.1 Cycle Sequencing Kit (Applied Biosystems, Foster City, CA, USA) and run on an ABI 3730 automated sequencer (Applied Biosystems).

### 3.3. Mitogenome Annotation and Analysis

All individuals are from the same clone, and the genetic variation was not found between isolates. So, one mitogenome was assembled. Sequences were assembled by the software SeqMan (DNAStar Inc., Madison, WI, USA). Sequence annotation was implemented using the blast tools on the NCBI web site [42]. The 13 PCGs and two ribosomal RNA genes were identified by sequence similarity with some published aphid mitogenomes (*A. pisum* NC_011594.1, *C. salicicola* NC_022682, *D. noxia* NC_022727 and *S. graminum* NC_006158.1). The nucleotide sequences of the PCGs were translated with the invertebrate mitogenome genetic code. The tRNAs were predicted by the tRNAscan-SE Search Server v.1.21 [43] using the default settings. Two tRNA genes (*tRNA-Asn* and *tRNA-Ser*(AGN))were not found by the tRNAscan-SE, but were identified by comparison with other aphids and edited by eyes. The A+T content and codon usage were calculated by the MEGA version 6.05 [44]. Strand asymmetry was calculated using the formulas GC skew = (G−C)/(G+C) and AT skew = (A−T)/(A+T) [45] for the strand encoding the majority of the protein-coding genes. The putative control region was detected for regions of potentially palindromes or inverted repeats with the Mfold web server [46].

### 3.4. Phylogenetic Analysis

For *M. keteleerifoliae*, one individual was used for molecular analysis. The multiple alignments of the concatenated 13 PCG nucleotide sequences for the 9 aphid mitogenomes, including 8 Aphididae species and one Phylloxeridae species as the outgroup (Table 1), were conducted with the MEGA version 6.05 [44] and then manually proofread. Alignments of individual genes were then concatenated after excluding the stop codons.

Maximum likelihood (ML) and Bayesian inference (BI) analyses were calculated using PHYML 3.0 [47] and MrBayes version 3.1.2 [48], respectively. The JModelTest 3.7 was implemented to select an appropriate nucleotide substitution model [49]. The GTR+I+G was the optimal model by the Bayesian information criterion (BIC) [50]. ML analyses were under the optimal substitution model from the JModelTest, and model parameter values were valued during the analyses. Nodal support of branches was evaluated by bootstrap analysis with 100 replicates. The Bayesian inference used two independent runs with 10,000,000 generations in each and four chains. Each chain was sampled every 1000 generations with a burn-in of 25%. Trees inferred prior to stationary were discarded as burn-in, and the remaining trees were constructed using a 50% majority-rule consensus tree with posterior probabilities.

## 4. Conclusion

This paper reports the complete mitogenome of the aphid*M. keteleerifoliae*, and compares the analysis to published aphid mitochondrial genomes. The results suggest that gene size, content, and base compositions are similar among Aphididae mitogenomes. Most tRNAs are folded into the classic clover-leaf structure, with the exception of *tRNA-Ser* (AGN). A tRNA isomer maybe identified in the mitogenomes of *M. keteleerifoliae* and *E. lanigerum*; whetherthe tRNA isomers in aphids exist needs further study. Phylogenetic reconstructions based on protein-coding genes showed Mindarinae at the basal position of Aphididae. Two tribes of Aphidinae were recovered with high statistical significance. Therefore, because only eight complete aphid mitogenomes were published, including *M. keteleerifoliae*, future studies with larger taxon sampling sizes are necessary.

## Supplementary Materials

Supplementary materials can be found at http://www.mdpi.com/1422-0067/16/12/26219/s1.
