# Supplementary material for: The Complete Mitochondrial Genome of Mindarus keteleerifoliae (Insecta: Hemiptera: Aphididae) and Comparison with Other Aphididae Insects
Source: Int J Mol Sci. 2015 Dec 17;16(12):30091–102. doi: 10.3390/ijms161226219 (PMC4691162; doi:10.3390/ijms161226219)
Supplement: Supplementary file 1 [file ijms-16-26219-s001.pdf]

Supplementary Materials: The Complete Mitochondrial Genome of *Mindarus keteleerifoliae* (Insecta: Hemiptera: Aphididae) and Comparison with Other Aphididae Insects

Yuan Wang, Jing Chen, Li-Yun Jiang and Ge-Xia Qiao

Table S1. Nucleotide composition of the *Mindarus keteleerifoliae* mitogenome.

Composition	A%	G%	C%	T%	A + T%	AT-Skew	GC-Skew	Length (bp)
Whole genome	44.6	6.0	9.6	39.9	84.5	0.056	−0.233	15,199
Protein-coding genes	35.9	7.7	8.4	47.9	83.4	−0.143	−0.041	10,946
First codon	40.8	5.7	5.9	48.0	88.4	−0.078	−0.021	3649
Second codon	38.2	8.9	8.9	44.0	82.2	−0.070	−0.002	3649
Third codon	28.8	8.6	10.4	52.0	81.0	−0.289	−0.094	3648
Protein-coding genes-J	38.6	6.7	10.3	44.4	83.0	−0.070	−0.209	6739
First codon	46.3	4.3	6.6	43.0	89.1	0.040	−0.208	2247
Second codon	40.0	9.3	11.7	39.0	79.0	0.014	−0.114	2246
Third codon	29.4	6.5	12.6	52.0	80.9	−0.274	−0.315	2246
Protein-coding genes-N	31.7	9.4	5.4	53.6	85.2	−0.257	0.269	4207
First codon	35.4	8.3	4.3	52.0	87.4	−0.191	0.311	1403
Second codon	27.9	11.9	6.9	53.0	81.2	−0.313	0.265	1402
Third codon	31.8	7.9	4.9	55	87.2	−0.270	0.233	1402
tRNA genes	45.0	6.2	7.9	40.9	85.9	0.048	−0.120	1478
tRNA genes-J	45.5	7.2	7.3	40.0	85.4	0.065	−0.007	941
tRNA genes-N	44.1	4.5	8.9	42.5	86.6	0.019	−0.333	537
rrnL gene	46.1	4.6	9.9	39.5	85.6	0.077	−0.366	1266
rrnS gene	42.4	5.5	10.2	42.0	84.4	0.005	−0.299	765
Control region	47.5	4.9	6.1	41.5	88.9	0.067	−0.105	687

Table S2. Codon usage in the mitogenome of *Mindarus keteleerifoliae.*

AA	Codon	Number	RSCU	AA	Codon	Number	RSCU
Phe（F)	**UUU**	**471**	**1.93**	Tyr(Y)	**UAU**	**156**	**1.91**
	UUC	17	0.07		UAC	7	0.09
Leu（L2)	**UUA**	**468**	**5.21**	His(H)	**CAU**	**49**	**1.81**
	UUG	24	0.27		CAC	5	0.19
Leu（L1)	CUU	16	0.18	Gln(Q)	**CAA**	**45**	**1.91**
	CUC	2	0.02		CAG	2	0.09
	**CUA**	**28**	**0.31**	Asn(N)	**AAU**	**283**	**1.9**
	CUG	1	0.01		AAC	15	0.1
Ile（I)	**AUU**	**460**	**1.87**	Lys(K)	**AAA**	**150**	**1.9**
	AUC	32	0.13		AAG	8	0.1
Met（M)	**AUA**	**322**	**1.91**	Asp(D)	**GAU**	**43**	**1.69**
	AUG	15	0.09		GAC	8	0.31
Val（V)	**GUU**	**39**	**2.17**	Glu(E)	**GAA**	**65**	**1.91**
	GUC	2	0.11		GAG	3	0.09
	GUA	30	1.67	Cys(C)	**UGU**	**25**	**1.72**
	GUG	1	0.06		UGC	4	0.28
Ser（S2)	UCU	86	2.32	Trp(W)	**UGA**	**76**	**1.79**
	UCC	12	0.32		UGG	9	0.21
	**UCA**	**107**	**2.88**	Arg(R)	CGU	12	1.23
	UCG	1	0.03		CGC	0	0
Pro（P)	**CCU**	**51**	**1.76**		**CGA**	**26**	**2.67**
	CCC	12	0.41		CGG	1	0.1
	**CCA**	**51**	**1.76**	Ser(S1)	AGU	29	0.78
	CCG	2	0.07		AGC	4	0.11
Thr（T)	ACU	45	1.7		**AGA**	**57**	**1.54**
	ACC	2	0.08		AGG	1	0.03
	**ACA**	**59**	**2.23**	Gly(G)	GGU	39	1.17
	ACG	0	0.00		GGC	0	0
Ala（A)	**GCU**	**38**	**2.34**		**GGA**	**82**	**2.47**
	GCC	2	0.12		GGG	12	0.36
	GCA	25	1.54	stop	UAA	0	0.00
	GCG	0	0.00		UAG	0	0.00

A total of 3637 codons from *Mindarus keteleerifoliae* were analyzed, excluding the stop codons. AA, amino acid; RSCU, Relative synonymous codon usage. Boldface represents each amino acid with the highest frequency codon and underlined letters refer to the codon that matches the tRNA anticodon.

Table S3. Primers used in this study.

No. Fragment	Mainly Locus	Primer ID	Nucleotide Sequence (5′-3′)	Reference
1	Co I & Co II	Lep F<br>A3772	ATTCAACCAATCATAAAGATATTGG<br>GAGACCATTACTTGCTTTCAGTCATCT	Foottit *et al.*, 2008 [47]<br>Normark *et al.*, 1996 [48]
2	Co II & Co III	CO2Af (mtDNA)<br>CO3WWRD (mtDNA)	AATCAYAGWTTTATRCCWATTCA<br>TCWCGAATWACATCWCGTCATCA	Ortiz-Rivas *et al.*, 2010 [49]
3	Co II & Co III	Co2f<br>Co3r	TAGATGACTGAAAAGCAAAG<br>GTAATCCATGAAATCCTGTT	Independent design
4	Co III & ND3	CO3WWRDre<br>nad3r	TGATGACGWGATGTWATTCGWGA<br>GATAGTTAGCAGCTTCTTTT	Independent design
5	ND3 & ND5	nd31<br>nd51	TGAATGTGGATTTGATCCATT<br>CCTTTTTCTTCTTGGTTWCCT	Independent design
6	ND5 & ND4	N5-J7572<br>nad4R	AAAGGGAATTTGAGCTCTTTTWGT<br>TCATGTTGAAGCTCCAATT	Simon *et al.*, 2006 [50]<br>Independent design
7	ND4L & CytB	N4-J8641x<br>CB-N10608	CCAGAAGAACATAAACCATG CCA<br>AGTARTGAWCCAAARTTTCA	Simon *et al.*, 2006 [50], modified
8	CytB & ND1 & 16S & 12S	CB1<br>12sai	TATGTACTACCATGAGGACAAATATC<br>AAACTAGGATTAGATACCCTATTAT	Simon *et al.*, 1994 [51]
9	12S & control region & ND2	12saire<br>ND2R②	ATAATAGGGTATCTAATCCTAGTTT<br>TAGGTGGTAAACCTATAATT	Simon *et al.*, 1994 [51]<br>Independent design
10	ND2& COI	ND2/CO IF<br>ND2/CO IR	CTCCACATGAATATTATTTGC<br>TTCCTGATCAAATACCAAATA	Independent design
